# Metabolic-dysfunction associated steatotic liver disease-related diseases, cognition and dementia: A two-sample mendelian randomization study

**DOI:** 10.1371/journal.pone.0297883

**Published:** 2024-02-29

**Authors:** Yao-Shuang Li, Yu-Ge Xia, Yan-Lan Liu, Wei-Ran Jiang, Hui-Na Qiu, Fan Wu, Jing-Bo Li, Jing-Na Lin

**Affiliations:** 1 Tianjin Union Medical Center, Tianjin Medical University, Tianjin, China; 2 Department of Endocrinology, Tianjin Union Medical Center, Nankai University Affiliated Hospital, Tianjin, China; 3 Geriatric Department, The Second Affiliated Hospital of Anhui University of Chinese Medicine, Hefei, Anhui, China; 4 Eastman Institute for Oral Health, University of Rochester Medical Center, Rochester, New York, United States of America; Kaohsiung Medical University Chung Ho Memorial Hospital, TAIWAN

## Abstract

**Background:**

The results of current studies on metabolic-dysfunction associated steatotic liver disease (MASLD)-related diseases, cognition and dementia are inconsistent. This study aimed to elucidate the effects of MASLD-related diseases on cognition and dementia.

**Methods:**

By using single-nucleotide polymorphisms (SNPs) associated with different traits of NAFLD (chronically elevated serum alanine aminotransferase levels [cALT], imaging-accessed and biopsy-proven NAFLD), metabolic dysfunction-associated steatohepatitis, and liver fibrosis and cirrhosis, we employed three methods of mendelian randomization (MR) analysis (inverse-variance weighted [IVW], weighted median, and MR-Egger) to determine the causal relationships between MASLD-related diseases and cognition and dementia. We used Cochran’s Q test to examine the heterogeneity, and MR-PRESSO was used to identify outliers (NbDistribution = 10000). The horizontal pleiotropy was evaluated using the MR-Egger intercept test. A leave-one-out analysis was used to assess the impact of individual SNP on the overall MR results. We also repeated the MR analysis after excluding SNPs associated with confounding factors.

**Results:**

The results of MR analysis suggested positive causal associations between MASLD confirmed by liver biopsy (p of IVW = 0.020, OR = 1.660, 95%CI = 1.082–2.546) and liver fibrosis and cirrhosis (p of IVW = 0.009, OR = 1.849, 95%CI = 1.169–2.922) with vascular dementia (VD). However, there was no evidence of a causal link between MASLD-related diseases and cognitive performance and other types of dementia (any dementia, Alzheimer’s disease, dementia with lewy bodies, and frontotemporal dementia). Sensitivity tests supported the robustness of the results.

**Conclusions:**

This two-sample MR analysis suggests that genetically predicted MASLD and liver fibrosis and cirrhosis may increase the VD risk. Nonetheless, the causal effects of NAFLD-related diseases on VD need more in-depth research.

## 1 Introduction

Because of its close association with metabolic abnormalities, the term "non-alcoholic fatty liver disease" has recently been replaced by "metabolic-dysfunction associated steatotic liver disease (MASLD)" [1]. MASLD is characterized by the abnormal accumulation of fat in the liver tissue in the absence of excessive intake of alcohol or other special causes. It ranges from simple hepatic steatosis, metabolic dysfunction-associated steatohepatitis (MASH), liver fibrosis, and hepatocellular carcinoma, depending on the stage of disease development. In recent years, the global prevalence of MASLD has continued to increase, and the adult prevalence was about 37% in 2019 [2], which poses a great threat to public health. MASLD is often accompanied by obesity and abnormal metabolism of blood lipids and glucose [3, 4], and has also been shown to be a risk factor for diabetes, hypertension, and cardiovascular and cerebrovascular diseases [5, 6]. Recently, it has been found that MASLD may also be associated with cognitive impairment and dementia. Since both MASLD and dementia are strongly associated with obesity, diabetes, and cardiovascular and cerebrovascular events [7, 8], it may be reasonable to speculate that there is an association between the two. Indeed, current research on MASLD-related diseases and cognitive impairment and dementia is limited and the results obtained are not consistent.

A longitudinal analysis from the Rotterdam Study showed that MASLD and fibrosis were not associated with either cognitive impairment or the risk of dementia [9], but results from another four-year longitudinal study supported a positive association between MASLD and risk of cognitive impairment [10]; the results of these two large, recently published longitudinal studies are conflicting. In addition, a recent meta-analysis of seven studies involving 891,562 subjects showed that MASLD was associated with an increased risk of cognitive impairment (OR = 1.44; 95%CI: 1.17–1.78; p = 0.001), but not with all-cause dementia (p = 0.341) or Alzheimer’s Disease (AD, p = 0.489) [11]. Overall, the current evidence is insufficient to elucidate the relationship between MASLD-related diseases and cognitive impairment and dementia. Moreover, due to the limitations of observational studies, a causal relationship between the two cannot be established and confounding factors are inevitable.

Mendelian randomization (MR) is an epidemiological research method that uses genetic variants to explore the causal relationship between exposure and outcome. Compared with traditional observational studies, MR can minimize the bias caused by confounding factors [12]. Several previous studies using MR have identified causal relationships between MASLD and some risk factors [13, 14], but so far no causal relationship between MASLD-related diseases and cognitive performance or dementia has been established.

Based on the Genome wide association study (GWAS) data from several large cohorts, this study aimed to elucidate the effects of MASLD-related diseases (MASLD, MASH, liver fibrosis, and cirrhosis) on global cognitive performance and risk of several types of dementia (any dementia, AD, vascular dementia [VD], dementia with lewy bodies [DLB], and frontotemporal dementia [FTD]) using two-sample MR analysis.

## 2 Methods

**Fig 1:**
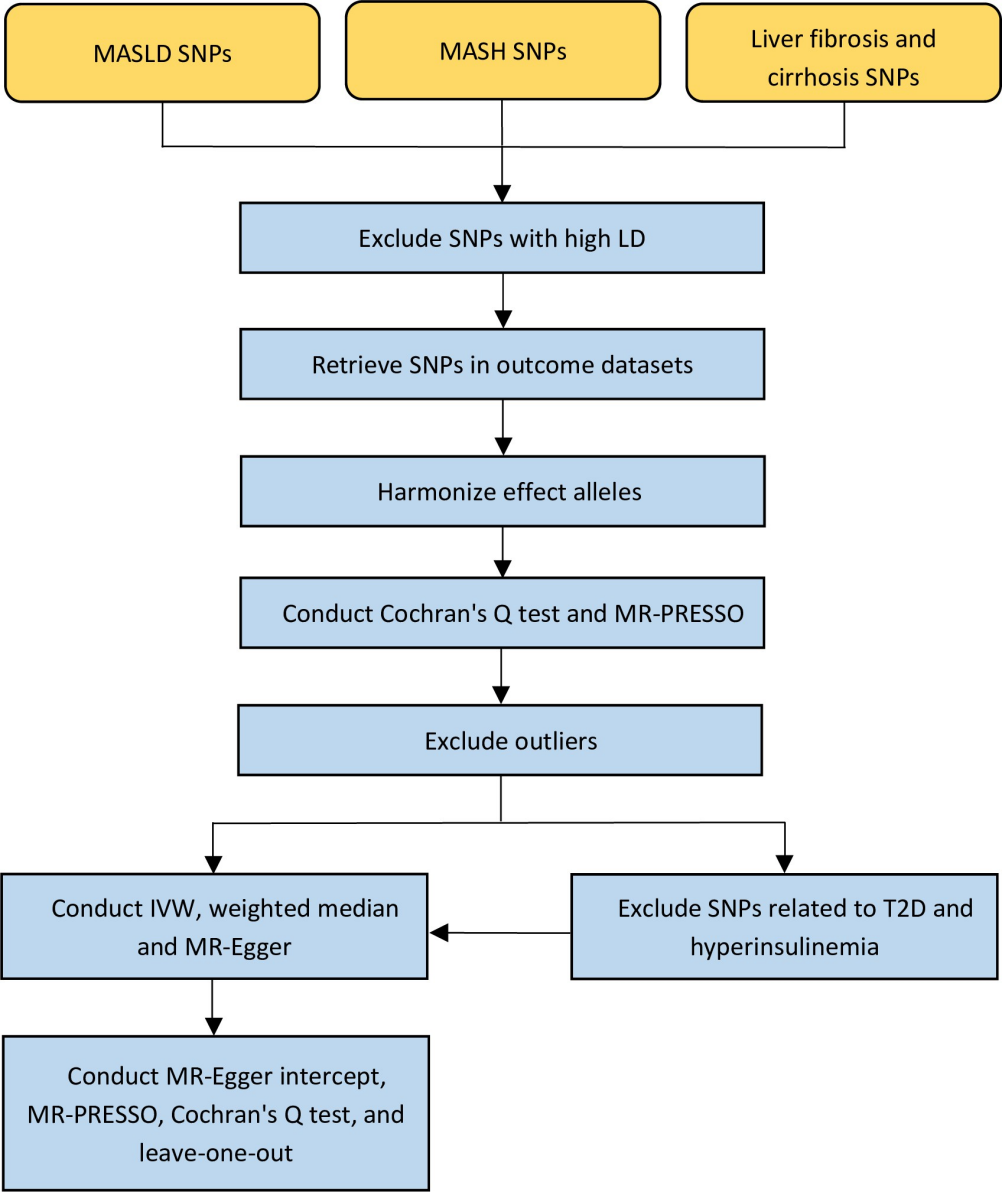
Flowchart of the Mendelian randomization study exploring the causal relationships between MASLD-related diseases, cognition, and dementia. MASLD, metabolic-dysfunction associated steatotic liver disease; MASH, metabolic dysfunction-associated steatohepatitis; SNPs, single-nucleotide polymorphisms; LD, linkage disequilibrium; T2D, type 2 diabetes.

Data used for analysis in this study were collected from pooled GWAS data that had been published, and therefore no additional ethical approval or informed consent was required. Fig 1 summarizes the flow of this study.

### 2.1 Data source for MASLD-related diseases and selection of instrumental variables (IVs)

Data on MASLD were obtained from a large-scale GWAS recently published by Vujkovic et al. [15], which referred to MASLD in terms of chronically unexplained elevated serum alanine aminotransferase (cALT). Specifically, an increase in ALT > 40 U/L in men or > 30 U/L in women at two time points at least 6 months apart within 2 years after exclusion of other liver diseases. The study reported 77 single-nucleotide polymorphisms (SNPs) that were significantly associated with cALT at the genome-wide level (p < 5×10^−8^) in an interracial discovery cohort (n = 218,595, 75.1% European Americans, 17.1% African Americans, 6.9% Hispanic Americans, and 0.9% Asian Americans). These SNPs were subsequently validated with two external cohorts (imaging-assessed liver fat [n = 44,289] and biopsis-proven MASLD [n = 63,969]), with 22 and 36 SNPs successfully replicated, respectively. Finally, there were 17 SNPs that were replicated in both validation cohorts. Therefore, four sets of IVs related to MASLD were used in the MR analysis: (1) 77 CALT-associated SNPs; (2) 22 SNPs associated with cALT in the imaging cohort; (3) 36 SNPs associated with cALT in the biopsy cohort; (4) 17 SNPs associated with cALT in both cohorts.

The SNPs associated with MASH and liver fibrosis and cirrhosis were derived from the FinnGen (https://www.finngen.fi/en/access_results), which contains aggregated GWAS data for a large number of European populations [16]. The summary data of MASH included 157 cases and 377,120 controls, and the summary data of liver fibrosis and cirrhosis included 1,841 cases and 366,450 controls. In order to obtain more IVs, we relaxed the correlation thresholds of MASH and liver fibrosis and cirrhosis to 5×10^−6^.

SNPs with linkage disequilibrium (LD, r^2^ > 0.001), incompatible SNPs and palindromic sequences with moderate minor allele frequency were excluded. F-statistics were also calculated to assess the strength of the IVs. F-statistics > 10 means that IVs can effectively represent exposure factors [17].

### 2.2 Data sources for cognitive performance and dementia

Summary statistics on cognitive performance was obtained from the Social Science Genetic Association Consortium (SSGAC). This dataset meta-analyzed GWAS data from the UK Biobank (n = 225,056, European ethnicity) and the Cognitive Genomics Consortium (COGENT, n = 35,298, European ethnicity) [18, 19] (S1 Table in S2 File). The ’g’ index was used here to assess overall cognitive function, which was calculated by integrating all cognitive assessment test scores for each individual [19]. Previous studies have shown that the comprehensive index ’g’ is basically consistent with the results of the original cognitive tests and can accurately reflect the global cognitive performance of subjects [20, 21].

Pooled GWAS data for AD was obtained from the Psychiatric Genomics Consortium (PGC). The data comes from a recent large-scale meta-analysis of GWAS from 13 cohorts by Wightman et al. [22] (S1 Table in S2 File). This study contained 90,338 late-onset AD (LOAD) cases (46,613 proxies) and 1,036,225 (318,246 proxies) controls of European ethnicity. Late age of onset poses difficulties in recruiting LOAD cases, so relatively young individuals were included in the study as proxy cases or controls, determined on the basis of their parents’ age and LOAD-related conditions.

The GWAS data of any dementia, VD, DLB, and FTD could be downloaded from https://gwas.mrcieu.ac.uk/. Participants in the any dementia included 5,933 cases and 212,859 controls of European ancestry, and participants in the VD included 98 cases and 211,300 controls of European ancestry. The GWAS data for DLB included 2,591 cases and 4,027 controls (European ethnicity) from 44 consortia [23]. The summary statistics on FTD included 515 cases and 2,509 controls (European ethnicity) from 45 clinical centers [24]. An overview of all the GWAS data included in this study is provided in Table 1.

**Table 1:** Overview of the data used in the mendelian randomization analysis.

	Phenotype	Sample size	Web source
Exposures	cALT (yes/no)	90,408 cases and 128,187 controls	www.ncbi.nlm.nih.gov/pmc/articles/PMC10024253/
	Liver fat evaluated by imaging (CT/MRI)	44,289	
	Biopsy‐confirmed MASLD (yes/no)	7,397 cases and 56,785 controls	
	MASH (yes/no)	157 cases and 377,120 controls	www.finngen.fi/en/access_results
	Liver fibrosis and cirrhosis (yes/no)	1,841 cases and 366,450 controls	
Outcomes	Cognitive performance (‘g’ index)	260,354	www.thessgac.org
	Any dementia (yes/no)	5,933 cases and 212,859 controls	https://gwas.mrcieu.ac.uk/datasets/finn-b-KRA_PSY_DEMENTIA/
	Alzheimer’s disease (yes/no)	90,338 cases and 1,036,225 controls	www.med.unc.edu/pgc/results-and-downloads
	Vascular dementia (yes/no)	98 cases and 211,300 controls	https://gwas.mrcieu.ac.uk/datasets/finn-b-VD_MX/
	Dementia with lewy bodies (yes/no)	2,591 cases and 4,027 controls	https://gwas.mrcieu.ac.uk/datasets/ebi-a-GCST90001390/
	Frontotemporal dementia (yes/no)	515 cases and 2,509 controls	https://gwas.mrcieu.ac.uk/datasets/ieu-b-43/

GWAS, genome-wide association study; cALT, chronically elevated serum alanine aminotransferase levels; CT, computed tomography; MRI, magnetic resonance imaging; MASLD, metabolic-dysfunction associated steatotic liver disease; MASH, metabolic dysfunction-associated steatohepatitis.

### 2.3 Statistical analysis

Three MR analysis methods (random-effect models of inverse-variance weighted [IVW], weighted median, and MR-Egger) were used to determine the causal relationships between MASLD-related diseases, cognitive performance, and dementia. IVW represents the primary analysis results, and this analysis is characterized by regression that does not consider the intercept term and uses the inverse of the squared outcome variance as the weight for fitting. As a supplementary estimation result, the assumption of MR-Egger analysis is that all IVs are pleiotropic, that is, the existence of intercept terms is considered in the regression, and the weighted median considers that half of the genetic variation is pleiotropic [25, 26]. Therefore, the combination of the three MR analysis methods is able to provide more robust outcome estimates.

Before MR analysis, we used Cochran’s Q test to determine the heterogeneity of IVs, and MR-PRESSO was used to identify potential outliers (NbDistribution = 10000). If outliers were present (p < 0.05), they were removed and then subjected to MR analysis [27]. Next, we also used multiple sensitivity analyses to ensure the reliability of our results. Horizontal pleiotropy was assessed using the MR-Egger intercept test. The presence of significant horizontal pleiotropy (p < 0.05) indicates that the genetic variants do not meet the basic condition for being IVs [28]. Leave-one-out analysis was used to evaluate the effect of individual SNP on the overall MR estimation results. We also calculated the overall statistical power (https://shiny.cnsgenomics.com/mRnd/), and the recommended value is > 80% [29].

Finally, in order to eliminate the effects of potential confounding factors, we also examined whether each tool SNP had genome-wide level association (p < 5×10^−8^) with key risk factors for cognitive impairment and dementia on the PhenoScanner (www.phenoscanner.medschl.cam.ac.uk) [30, 31], including cerebrovascular disease, hypertension, heart disease (atrial fibrillation, arrhythmia and cardiac arrest), atherosclerosis, cerebral amyloid angiopathy, type 2 diabetes (T2D), hyperinsulinemia, traumatic brain injury, epilepsy, depression, respiratory diseases, anemia, hearing disorders, sleep disorders, brain-derived neurotrophic factor, homocysteine, educational attainment, physical activity, diet, smoking, and alcohol drinking [32, 33].

TwoSampleMR (0.4.9) and MR-PRESSO (1.0) packages of R software (4.3.0) were used to perform all analyses. Two-sided p values of less than 0.05 were considered to indicate statistical significances.

## 3 Results

### 3.1 Causal relationship between MASLD-related diseases and cognitive performance

The cALT-related SNPs in LD were removed and then merged and harmonized with the outcome data of cognitive performance, resulting in 45 IVs with F-statistics ranging from 35 to 167 that could be used for further analysis (S2 Table in S2 File). The following Cochran’s Q test suggested the presence of heterogeneity: p of IVW = 1.02e-07. Consistent with this finding, MR-PRESSO test also suggested the presence of outliers (p < 1e-04, rs10883451, rs174535 and rs5117). Thus, we performed MR analysis after removing the three outliers (S2 Table in S2 File), and the results suggested no causal relationship between genetically predicted MASLD and global cognitive performance (p of IVW = 0.847, Table 2). Similarly, we performed these MR analyses with the use of three other sets of IVs representing MASLD (S2 Table in S2 File). According to the results of heterogeneity test and MR-PRESSO analysis, we performed MR analysis after removing outliers (rs5117 in imaging-assessed cohort, rs10883451 and rs5117 in biopsy-confirmed cohort, and rs5117 in these two cohorts). The results were consistent with our previous findings, and all suggested no causal relationship between genetically predicted MASLD and global cognitive performance (all p values were greater than 0.05, Table 2).

**Table 2:** Mendelian randomization analysis of MASLD-related diseases and cognitive performance.

IVs	Analysis	β	95%CI	P value
cALT (42 SNPs)	IVW	-0.002	-0.018, 0.015	0.847
	MR Egger	0.005	-0.023, 0.033	0.739
	Weighted median	0.000	-0.022, 0.023	0.976
	MR-Egger intercept	\	\	0.581
Imaging (16 SNPs)	IVW	-0.024	-0.018, 0.015	0.847
	MR Egger	-0.036	-0.023, 0.033	0.739
	Weighted median	-0.016	-0.022, 0.023	0.976
	MR-Egger intercept	\	\	0.641
Biopsy (25 SNPs)	IVW	-0.002	-0.012, 0.007	0.620
	MR Egger	-0.003	-0.016, 0.010	0.635
	Weighted median	-0.002	-0.011, 0.008	0.722
	MR-Egger intercept	\	\	0.853
Imaging and biopsy (13 SNPs)	IVW	-0.003	-0.013, 0.008	0.594
	MR Egger	-0.007	-0.021, 0.008	0.369
	Weighted median	-0.002	-0.012, 0.007	0.620
	MR-Egger intercept	\	\	0.436
MASH (3 SNPs)	IVW	-0.004	-0.015,0.008	0.540
	MR Egger	-0.033	-0.063,-0.002	0.283
	Weighted median	-0.001	-0.009,0.009	0.949
	MR-Egger intercept	\	\	0.304
Liver fibrosis and cirrhosis (12 SNPs)	IVW	0.003	-0.007,0.013	0.574
	MR Egger	0.003	-0.015,0.022	0.719
	Weighted median	0.004	-0.007,0.015	0.457
	MR-Egger intercept	\	\	0.942

MASLD, metabolic-dysfunction associated steatotic liver disease; IVs, instrumental variables; CI, confidence interval; cALT, chronically elevated serum alanine aminotransferase level; SNPs, single-nucleotide polymorphisms; IVW, inverse variance weighted; MASH, metabolic dysfunction-associated steatohepatitis.

After data processing, we obtained 3 MASH IVs and 12 liver fibrosis and cirrhosis IVs that could be used for MR analysis (S2 Table in S2 File). Cochran’s Q test showed no heterogeneity: p of IVW = 0.102 or 0.060. MR-PRESSO tests also showed no outliers. The results of the subsequent MR analysis showed that neither genetically predicted MASH nor liver fibrosis and cirrhosis was associated with global cognitive performance (all p values were greater than 0.05, Table 2). In addition, MR-Egger intercept tests showed no horizontal pleiotropy (p > 0.05; Table 2). No single SNP strongly violated the overall effect of MASLD-related diseases on cognitive performance in leave-one-out sensitivity analyses (S1-S3 Figs in S1 File).

Finally, in order to exclude the possible influence of potential confounders, based on the results searched online (www.phenoscanner.medschl.cam.ac.uk), we further excluded the SNPs associated with T2D (rs13389219, rs17036160, rs2943652, rs56094641 and rs58542926) and fasting insulin (rs4841132) in the c-ALT IVs. Results of MR analysis still showed no causal relationship between MASLD and cognitive performance (p of IVW = 0.996, p of MR-Egger = 0.375, p of weighted median = 0.958). Similar results were obtained after removing these confounding SNPs from the other three sets of IVs. We also found a T2D-related SNP (rs1802295) in the IVs of liver fibrosis and cirrhosis. After excluding this SNP, the results of the MR analysis were consistent (p of IVW = 0.846, p of MR-Egger = 0.454, p of weighted median = 0.475).

### 3.2 Causal relationship between MASLD-related diseases and any dementia

After removing SNPs in LD and merging and harmonizing them with the outcome data on any dementia, the numbers of MASLD-related diseases IVs that were available for further analyses were 52, 16, 28, 13, 3, and 14, respectively (S3 Table in S2 File). Cochran’s Q test suggested no heterogeneity, and MR-PRESSO analysis showed no outliers (p > 0.05). MR analysis showed no causal association between genetically predicted MASLD-related diseases and any dementia (all p values were greater than 0.05, Table 3). Sensitivity analysis showed no horizontal pleiotropy (p > 0.05, Table 3) and no single SNP significantly affected overall MR results (S4-S6 Figs in S1 File).

**Table 3:** Mendelian randomization analysis of MASLD-related diseases and any dementia.

IVs	Analysis	OR	95%CI	P value
cALT (52 SNPs)	IVW	0.973	0.892,1.061	0.535
	MR Egger	1.051	0.907,1.219	0.510
	Weighted median	0.980	0.850,1.129	0.776
	MR-Egger intercept	\	\	0.212
Imaging (16 SNPs)	IVW	0.833	0.617,1.126	0.235
	MR Egger	0.914	0.605,1.380	0.675
	Weighted median	0.910	0.637,1.301	0.606
	MR-Egger intercept	\	\	0.532
Biopsy (28 SNPs)	IVW	0.963	0.908,1.020	0.199
	MR Egger	1.001	0.925,1.084	0.981
	Weighted median	0.974	0.913,1.039	0.420
	MR-Egger intercept	\	\	0.176
Imaging and biopsy (13 SNPs)	IVW	0.963	0.910,1.019	0.189
	MR Egger	0.982	0.907,1.062	0.654
	Weighted median	0.976	0.914,1.042	0.460
	MR-Egger intercept	\	\	0.511
MASH (3 SNPs)	IVW	0.980	0.928,1.035	0.470
	MR Egger	0.974	0.754,1.258	0.874
	Weighted median	0.990	0.933,1.050	0.732
	MR-Egger intercept	\	\	0.971
Liver fibrosis and cirrhosis (14 SNPs)	IVW	1.039	0.971,1.102	0.265
	MR Egger	0.986	0.838,1.159	0.866
	Weighted median	1.021	0.945,1.103	0.603
	MR-Egger intercept	\	\	0.492

MASLD, metabolic-dysfunction associated steatotic liver disease; IVs, instrumental variables; OR, odds ratio; CI, confidence interval; cALT, chronically elevated serum alanine aminotransferase level; SNPs, single-nucleotide polymorphisms; IVW, inverse variance weighted; MASH, metabolic dysfunction-associated steatohepatitis.

In addition, by searching for the second phenotype, we identified SNPs associated with T2D (rs13389219, rs17036160, rs2943652, rs56094641 and rs58542926), fasting insulin (rs4841132) and hypertension (rs7653249) in the MASLD cohort, and SNPs associated with T2D (rs1802295 and rs8100204) in the liver fibrosis and cirrhosis cohort. After excluding these potentially confounding SNPs, the results of the MR analysis still indicated no causal association (p > 0.05).

### 3.3 Causal relationship between MASLD-related diseases and AD

The numbers of MASLD-related diseases IVs that were available for MR analyses were 38, 14, 22, 11, 2, and 12, respectively (S4 Table in S2 File). Neither Cochran’s Q test nor MR-PRESSO analysis suggested heterogeneity or outliers in these IVs (p > 0.05). When MR analyses were performed using the first set of IVs (associated with cALT, n = 38), the third set (associated with cALT in the biopsy cohort, n = 22), and the fourth set (associated with cALT in both the biopsy and imaging cohorts, n = 11), the p values of IVW were greater than 0.05, which represented the results of the primary analysis (25), as well as for MR-Egger. Therefore, despite the small p values of weighted median (p = 0.026, 0.039 and 0.039, respectively), the overall results of MR analysis suggested that there was no causal relationship between MASLD and AD (Table 4). However, when MR analysis was conducted using the second set of IVs (associated with cALT in the imaging cohort, n = 14), the results indicated a significant association between genetically predicted MASLD and AD (p of IVW = 0.049, p of MR-Egger = 0.181, p of weighted median = 0.048, Table 4). On the other hand, since the number of IVs related to MASH was less than 3, we could only conduct IVW analysis, and the result showed that there was no causal relationship between MASH and AD (p of IVW = 0.095, Table 4). There was also no evidence of a causal relationship between liver fibrosis and cirrhosis and AD (p of IVW = 0.179, Table 4). MR-Egger intercept test did not suggest any pleiotropy (p > 0.05, Table 4). Leave-one-out analysis also indicated that no single SNP strongly violated the global effect of MASLD-related diseases on AD (S7 and S8 Figs in S1 File).

**Table 4:** Mendelian randomization analysis of MASLD-related diseases and AD.

IVs	Analysis	OR	95%CI	P value
cALT (38 SNPs)	IVW	0.876	0.748,1.027	0.103
	MR Egger	0.777	0.594,1.018	0.076
	Weighted median	0.761	0.598,0.968	0.026*
	MR-Egger intercept	\	\	0.289
Imaging (14 SNPs)	IVW	0.576	0.332,0.998	0.049*
	MR Egger	0.572	0.264,1.237	0.181
	Weighted median	0.542	0.296,0.997	0.048*
	MR-Egger intercept	\	\	0.979
Imaging after corrected (9 SNPs)	IVW	0.554	0.261,1.176	0.124
	MR Egger	0.546	0.184,1.622	0.312
	Weighted median	0.516	0.272,0.980	0.043*
	MR-Egger intercept	\	\	0.971
Biopsy (22 SNPs)	IVW	0.913	0.833,1.001	0.052
	MR Egger	0.876	0.773,0.994	0.053
	Weighted median	0.890	0.890,0.796	0.039*
	MR-Egger intercept	\	\	0.360
Imaging and biopsy (11 SNPs)	IVW	0.909	0.824,1.003	0.058
	MR Egger	0.867	0.757,0.994	0.070
	Weighted median	0.891	0.798,0.994	0.039*
	MR-Egger intercept	\	\	0.350
MASH (2 SNPs)	IVW	0.909	0.814,1.016	0.095
Liver fibrosis and cirrhosis (12 SNPs)	IVW	0.898	0.768,1.050	0.179
	MR Egger	0.779	0.583,1.041	0.123
	Weighted median	0.839	0.724,0.972	0.020*
	MR-Egger intercept	\	\	0.283

MASLD, metabolic-dysfunction associated steatotic liver disease; AD, Alzheimer’s disease; IVs, instrumental variables; OR, odds ratio; CI, confidence interval; cALT, chronically elevated serum alanine aminotransferase level; SNPs, single-nucleotide polymorphisms; IVW, inverse variance weighted; MASH, metabolic dysfunction-associated steatohepatitis.

We then repeated MR analysis with the exclusion of five T2D-related SNPs (rs13389219, rs17036160, rs2943652, rs56094641 and rs58542926) in the MASLD IVs and one T2D-related SNP (rs1802295) in the liver fibrosis and cirrhosis IVs, and all results showed no causal relationship between MASLD-related diseases and risk of AD (p > 0.05). This meant that the relationship between image-verified MASLD and AD became insignificant (p of IVW = 0.124, Table 4), indicating that the previous positive results were due to the confounding factor. MR-Egger intercept test showed no significant horizontal pleiotropy (p > 0.05, Table 4).

### 3.4 Causal relationship between MASLD-related diseases and VD

After processing the data, there were 52, 16, 28, 13, 3, 14 MASLD-related diseases IVs that could be used for MR analysis. The Cochran’s Q test suggested the presence of heterogeneity in the c-ALT cohort: p of IVW = 0.039, and MR-PRESSO test also suggested one outlier (p = 0.030, rs1658943). Thus, we performed MR analysis after excluding this outlier (S5 Table in S2 File). The IWV, which represents the primary MR result, showed no causal association between MASLD and MASH for VD (p > 0.05, Table 5), although the P-values obtained by other analyses were less than 0.05. However, the MR analysis indicated a significant association between genetically predicted liver fibrosis and cirrhosis and VD (p of IVW = 0.009, p of MR-Egger = 0.429, p of weighted median = 0.021, Table 5). Sensitivity analysis showed no significant intercept (p > 0.05; Table 5) and no single SNP significantly affected overall MR estimates (S9-S11 Figs in S1 File).

**Table 5:** Mendelian randomization analysis of MASLD-related diseases and VD.

IVs	Analysis	OR	95%CI	P value
cALT (51 SNPs)	IVW	1.431	0.734,2.794	0.293
	MR Egger	3.261	1.045,10.172	0.047*
	Weighted median	2.331	0.863,6.293	0.095
	MR-Egger intercept	\	\	0.089
Imaging (16 SNPs)	IVW	2.950	0.359,24.257	0.314
	MR Egger	15.471	0.860,278.204	0.084
	Weighted median	39.595	2.515,623.278	0.009*
	MR-Egger intercept	\	\	0.123
Biopsy (28 SNPs)	IVW	1.368	0.913,2.051	0.129
	MR Egger	1.688	0.962,2.961	0.079
	Weighted median	1.941	1.193,3.159	0.008*
	MR-Egger intercept	\	\	0.301
Biopsy after corrected (23 SNPs)	IVW	1.660	1.082,2.546	0.020*
	MR Egger	2.105	1.166,3.801	0.022*
	Weighted median	1.950	1.215,3.129	0.006*
	MR-Egger intercept	\	\	0.269
Imaging and biopsy (13 SNPs)	IVW	1.243	0.834,1.852	0.286
	MR Egger	1.755	1.011,3.049	0.071
	Weighted median	1.467	0.904,2.382	0.121
	MR-Egger intercept	\	\	0.108
MASH (3 SNPs)	IVW	1.406	0.710,2.782	0.328
	MR Egger	0.156	0.027,0.895	0.285
	Weighted median	1.580	0.994,2.511	0.053
	MR-Egger intercept	\	\	0.240
Liver fibrosis and cirrhosis (14 SNPs)	IVW	1.849	1.169,2.922	0.009*
	MR Egger	1.596	0.521,4.893	0.429
	Weighted median	1.958	1.106,3.465	0.021*
	MR-Egger intercept	\	\	0.782

MASLD, metabolic-dysfunction associated steatotic liver disease; VD, vascular dementia; IVs, instrumental variables; OR, odds ratio; CI, confidence interval; cALT, chronically elevated serum alanine aminotransferase level; SNPs, single-nucleotide polymorphisms; IVW, inverse variance weighted; MASH, metabolic dysfunction-associated steatohepatitis.

Next, we repeated MR analysis with the exclusion of SNPs associated with T2D (rs13389219, rs17036160, rs2943652, rs56094641 and rs58542926), fasting insulin (rs4841132) and hypertension (rs7653249) in the MASLD IVs and two T2D-related SNPs (rs1802295 and rs8100204) in the liver fibrosis and cirrhosis IVs. Unlike before, we found that the causal association between MASLD confirmed by liver biopsy and VD became significant (p of IVW = 0.020, p of MR-Egger = 0.022, p of weighted median = 0.006, Table 5). MR-Egger intercept test also proved that the result was reliable (p > 0.05, Table 5). The associations between the other three sets of MASLD-related IVs and VD were also more significant than before, although still not statistically significant (p of IVW = 0.056, 0.051, and 0.050; S6 Table in S2 File). The MR results for MASH and liver fibrosis and cirrhosis and VD remained the same as before.

### 3.5 Causal relationship between MASLD-related diseases and DLB

The numbers of MASLD-related diseases IVs that were available for further MR analyses were 49, 16, 27, 12, 3, and 13, respectively (S7 Table in S2 File). Cochran’s Q test suggested no heterogeneity, and MR-PRESSO analysis showed no outliers (p > 0.05). MR analysis showed no causal association between genetically predicted MASLD-related diseases and DLB (all p values were greater than 0.05, Table 6). Sensitivity analysis showed no horizontal pleiotropy (p > 0.05, Table 6) and no single SNP significantly affected overall MR results (S12-14 Figs in S1 File).

**Table 6:** Mendelian randomization analysis of MASLD-related diseases and DLB.

IVs	Analysis	OR	95%CI	P value
cALT (49 SNPs)	IVW	0.884	0.758,1.031	0.116
	MR Egger	0.915	0.700,1.967	0.520
	Weighted median	0.965	0.771,1.208	0.757
	MR-Egger intercept	\	\	0.758
Imaging (16 SNPs)	IVW	0.668	0.392,1.139	0.139
	MR Egger	0.487	0.235,1.011	0.074
	Weighted median	0.607	0.319,1.154	0.128
	MR-Egger intercept	\	\	0.234
Biopsy (27 SNPs)	IVW	0.968	0.882,1.061	0.484
	MR Egger	0.918	0.807,1.044	0.203
	Weighted median	0.916	0.812,1.034	0.156
	MR-Egger intercept	\	\	0.258
Imaging and biopsy (12 SNPs)	IVW	0.941	0.850,1.041	0.239
	MR Egger	0.923	0.800,1.064	0.295
	Weighted median	0.914	0.816,1.025	0.124
	MR-Egger intercept	\	\	0.706
MASH (3 SNPs)	IVW	0.945	0.857,1.041	0.249
	MR Egger	1.114	0.756,1.639	0.682
	Weighted median	0.956	0.854,1.069	0.429
	MR-Egger intercept	\	\	0.547
Liver fibrosis and cirrhosis (13 SNPs)	IVW	0.961	0.875,1.055	0.400
	MR Egger	0.906	0.767,1.070	0.269
	Weighted median	0.948	0.833,1.080	0.422
	MR-Egger intercept	\	\	0.421

MASLD, metabolic-dysfunction associated steatotic liver disease; DLB, dementia with lewy bodies; IVs, instrumental variables; OR, odds ratio; CI, confidence interval; cALT, chronically elevated serum alanine aminotransferase level; SNPs, single-nucleotide polymorphisms; IVW, inverse variance weighted; MASH, metabolic dysfunction-associated steatohepatitis.

We also conducted MR analysis with the exclusion of SNPs associated with T2D (rs13389219, rs17036160, rs2943652, rs56094641 and rs58542926), fasting insulin (rs4841132) and hypertension (rs7653249) in the MASLD IVs and two T2D-related SNPs (rs1802295 and rs8100204) in the liver fibrosis and cirrhosis IVs. The results were consistent with the previous results and still showed no significant association between MASLD-related diseases and DLB.

### 3.6 Causal relationship between MASLD-related diseases and FTD

After processing the data, there were 4, 1, 1, 2 MASLD-related diseases IVs that could be used for MR analysis of FTD. Neither Cochran’s Q test nor MR-PRESSO analysis suggested heterogeneity or outliers in these IVs (p > 0.05). Since the numbers of IVs in the imaging, biopsy and liver fibrosis and cirrhosis cohorts were less than 3, we could only conduct wald ratio or IVW analysis, and the result showed that there was no causal relationship between MASLD-related diseases and FTD (all p values were greater than 0.05, Table 7). Sensitivity analysis suggested that there was no significant intercept (p > 0.05, Table 7) and no single SNP significantly affected overall MR results (S15 Fig in S1 File). After removing one T2D-related SNP (rs1802295) in the liver fibrosis and cirrhosis cohort, the MR analysis still showed no significant correlation.

**Table 7:** Mendelian randomization analysis of MASLD-related diseases and FTD.

IVs	Analysis	OR	95%CI	P value
cALT (4 SNPs)	IVW	0.879	0.236,3.273	0.848
	MR Egger	0.818	0.002,289.690	0.953
	Weighted median	0.863	0.207,3.596	0.840
	MR-Egger intercept	\	\	0.983
Imaging (1 SNP)	Wald ratio	0.036	0.000,123097.8	0.665
Biopsy (1 SNP)	Wald ratio	0.485	0.063,3.733	0.487
Liver fibrosis and cirrhosis (2 SNPs)	IVW	0.769	0.438,1.350	0.360

MASLD, metabolic-dysfunction associated steatotic liver disease; FTD, frontotemporal dementia; IVs, instrumental variables; OR, odds ratio; CI, confidence interval; cALT, chronically elevated serum alanine aminotransferase level; SNPs, single-nucleotide polymorphisms; IVW, inverse variance weighted.

## 4 Discussion

By using the genes of MASLD-associated diseases as IVs, the results of MR analysis suggest causal associations between MASLD confirmed by liver biopsy and liver fibrosis and cirrhosis with VD. Genetically predicted MASLD and liver fibrosis and cirrhosis may increase the risk of VD. However, we did not find a causal association between MASLD-related diseases and cognitive performance and other types of dementia.

The MR analysis showed that MASLD (based on cALT in the imaging cohort) was a protective factor for AD, but this association disappeared when the confounder T2D was excluded. We speculated that this result was mainly driven by T2D. To verify the conjecture, we performed a leave-one-out analysis. The result showed that there were three confounding SNPs that might have great impacts on the overall MR result: rs2943652, rs56094641 and rs58542926. The result of MR analysis was no longer significant after the removal of these SNPs (S16 Fig in S1 File). In fact, extra copies of the effecting alleles in the three SNPs were associated with a reduced risk of T2D [34]. So overall, our results did not prove a causal link between MASLD (based on cALT in the imaging cohort) and AD.

The current findings suggest that MASLD and liver fibrosis and cirrhosis are associated with VD risk, which support some recent studies. Evidence from a recent large matched cohort study involving more than 30,000 people showed that MASLD was associated with increased risk of VD. The diagnosis of MASLD is based on the International Classification of Diseases code from the National Patient Registry in Sweden [35]. Another large cross-sectional study from rural China also suggested that moderate and severe MASLD, as estimated by abdominal ultrasonography, was associated with VD [36]. Nevertheless, another prospective cohort study with a median follow-up of 15.5 years showed that MASLD, as estimated by fatty liver index, was not associated with either the risk of incident dementia or cognitive decline [9]. The different diagnostic methods of MASLD (e.g., fatty liver index, abdominal ultrasound) and different cognitive function measures (e.g., various cognitive function tests) used in these studies may account for the inconsistent results. In addition, even with a prospective study design, there will inevitably be inverse associations and confounding factors that can interfere with the results. Therefore, the MR design of our study can effectively overcome the limitations of observational studies to reveal whether there is a causal relationship between MASLD and cognitive ability and risk of dementia. Compared with previous MASLD-related MR analysis [37], the SNPs associated with MASLD at the genome-wide level used in our study were collected from gene summary data of a larger population with sufficient statistical strength. In addition, we used four sets of IVs associated with different phenotypes of MASLD, including liver biopsy-proven MASLD, which is the gold standard for MASLD diagnosis, thereby ensuring that our MR results are more reliable and robust. To ensure the rigor of the study, we also searched the website for SNPs associated with more than a dozen confounding factors and performed sensitivity analysis after excluding confounding SNPs.

Our results suggest that MASLD-associated diseases are associated with VD, but not with other types of dementia (including AD, DLB and FTD). This may be because the association between MASLD and dementia is primarily mediated by vascular lesions. In fact, metabolic disorders in MASLD patients can lead to a variety of vascular damage, such as atherosclerosis, vascular endothelial dysfunction, reduced blood flow [38]. The clinical manifestations of various vascular diseases (stroke, coronary heart disease, hypertension, etc.) are also closely related to MASLD [39]. In addition, there is evidence that patients with MASLD have decreased middle cerebral artery blood flow, and long-term cerebral hypoperfusion can induce microvascular lesions and accelerate the occurrence of cognitive decline and dementia [40]. Besides, a study has shown that biomarkers of fibrosis are better predictors of dementia risk than traditional cardiometabolic risk factors [35]. In severe hepatic steatosis or fibrosis, the systemic chronic inflammatory state caused by adipokine dysfunction may be one of the main causes of dementia [41]. Previous studies have shown that inflammation can significantly accelerate cognitive decline [42, 43].

To the best of our knowledge, this is the first study to explore the causal relationship between genetically predicted MASLD and global cognitive performance and dementia risk using the two-sample MR analysis. The main advantage of this study is that the MR design can effectively simulate the randomized controlled trial and minimize the influence of confounding bias, because the SNPs used as IVs in the study are randomly assigned to the offspring. At the same time, MR studies can avoid the possible reverse causality in observational studies. Given the high prevalence of MASLD in the population and the significant disease burden from cognitive impairment and dementia, our findings may have implications for primary care policy making to the extent that enhanced screening for VD in patients with genetically determined MASLD may be useful.

However, there are some limitations to this study. First, the cALT we used is not a perfect biomarker for MASLD [44], and therefore some MASLD susceptibility genes unrelated to cALT may be missed. But we also used imaging-based and biopsy-proven MASLD-associated SNPs as IVs, which also ensured the reliability of the current study method. Second, due to the limitations of the summary-level dataset, we were unable to further analyze the causal relationship between MASLD-related diseases and function in different cognitive domains.

## 5 Conclusions

This two-sample MR analysis suggests that genetically predicted MASLD and liver fibrosis and cirrhosis may increase the VD risk. Anyway, more prospective studies are needed to prove this link in the future.

## Supporting information

S1 File Leave-one-out analysis for MASLD-related diseases and cognitive performance and dementia. (PDF)

S2 File Description of outcome data and construction of instrumental variables. (XLSX)

## List of abbreviations

MASLD: Metabolic-dysfunction associated steatotic liver disease
MASH: Metabolic dysfunction-associated steatohepatitis
AD: Alzheimer’s Disease
VD: Vascular dementia
DLB: Dementia with lewy bodies
FTD: Frontotemporal dementia
SNP: Single-nucleotide polymorphism
MR: Mendelian randomization
IVW: Inverse-variance weighted
GWAS: Genome wide association study
IV: Instrumental variable
cALT: Chronically unexplained elevated serum alanine aminotransferase
LD: Linkage disequilibrium
LOAD: Late-onset Alzheimer’s Disease
T2D: Type 2 diabetes
