# Supplementary material for: Metabolic-dysfunction associated steatotic liver disease-related diseases, cognition and dementia: A two-sample mendelian randomization study
Source: PLoS One. 2024 Feb 29;19(2):e0297883. doi: 10.1371/journal.pone.0297883 (PMC10903857; doi:10.1371/journal.pone.0297883)

Supplementary Figures

- **Figures S1-S3. Leave-one-out analysis for MASLD-related diseases and cognitive performance.**
- **Figures S4-S6. Leave-one-out analysis for MASLD-related diseases and any dementia.**
- **Figures S7 and S8. Leave-one-out analysis for MASLD-related diseases and AD.**
- **Figures S9-S11. Leave-one-out analysis for MASLD-related diseases and VD.**
- **Figures S12-S14. Leave-one-out analysis for MASLD-related diseases and DLB.**
- **Figure S15. Leave-one-out analysis for MASLD (based on cALT) and FTD.**
- **Figure S16. Leave-one-out analysis for MASLD (based on image-supported cALT) and AD.**

Abbreviations: MASLD, metabolic-dysfunction associated steatotic liver disease; AD, Alzheimer's disease; VD, vascular dementia; DLB, dementia with lewy bodies; cALT, chronically elevated serum alanine aminotransferase level; FTD, frontotemporal dementia.

Figure S1. Leave-one-out analysis for MASLD (based on cALT) and cognitive performance

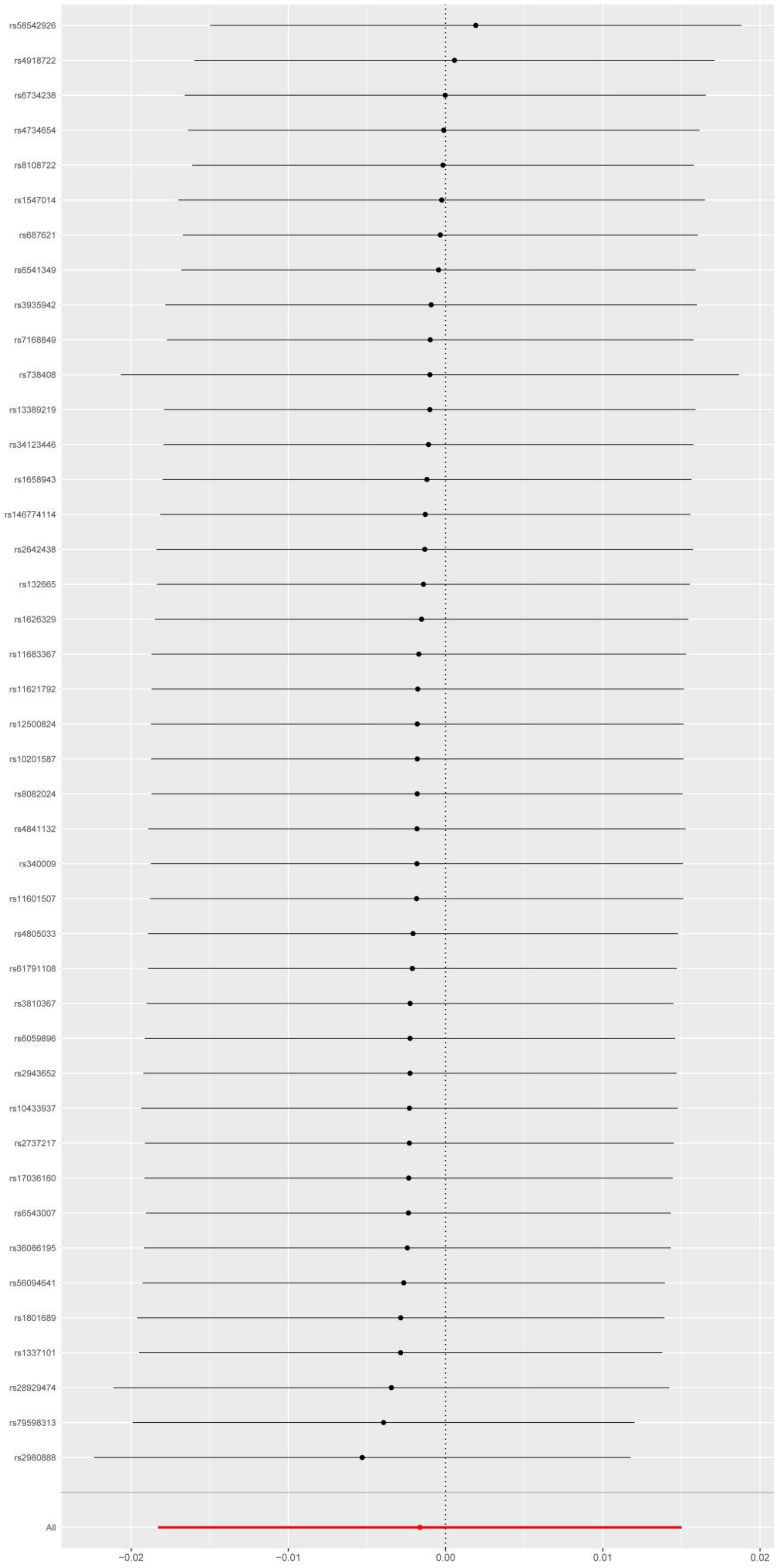

Figure S2. Leave-one-out analysis for MASH and cognitive performance

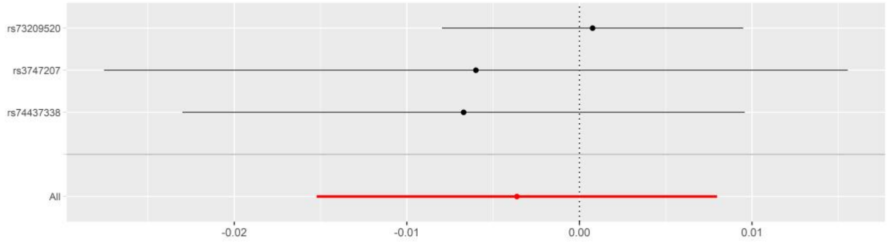

Figure S3. Leave-one-out analysis for liver fibrosis and cirrhosis and cognitive performance

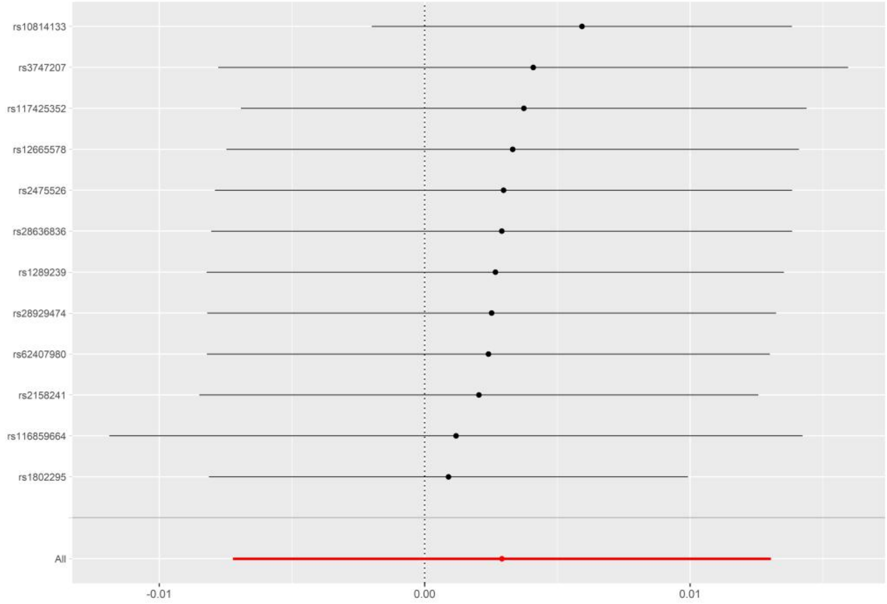

Figure S4. Leave-one-out analysis for MASLD (based on cALT) and any dementia

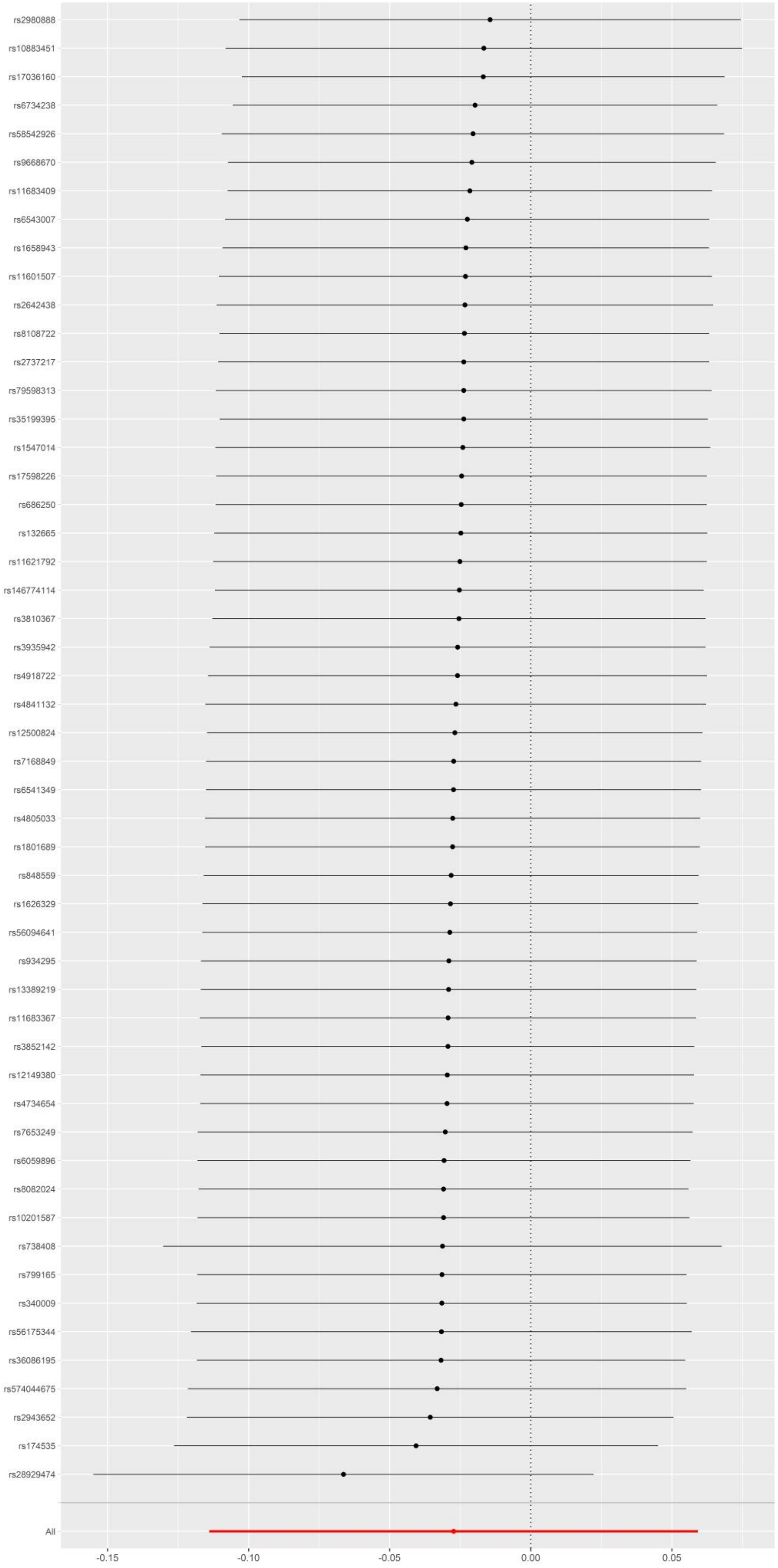

Figure S5. Leave-one-out analysis for MASH and any dementia

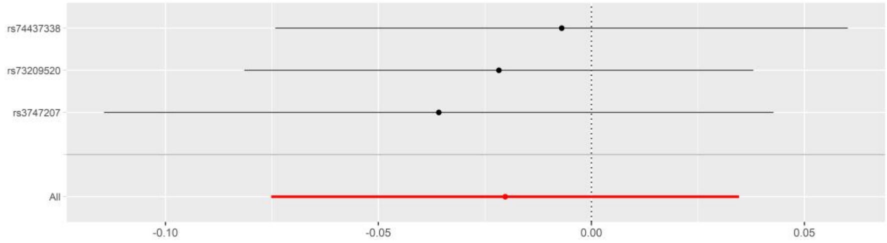

Figure S6. Leave-one-out analysis for liver fibrosis and cirrhosis and any dementia

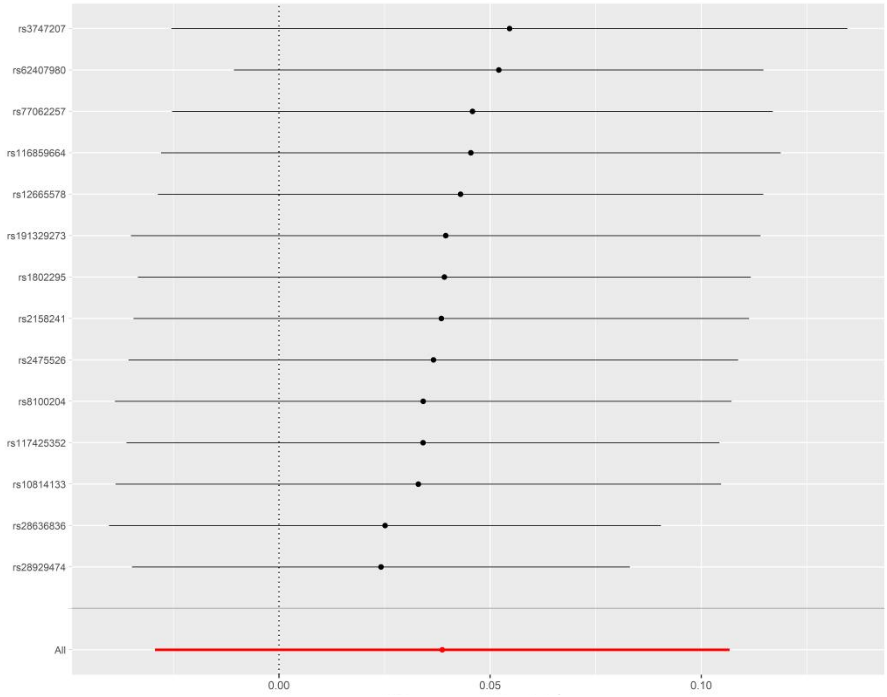

Figure S7. Leave-one-out analysis for MASLD (based on cALT) and AD

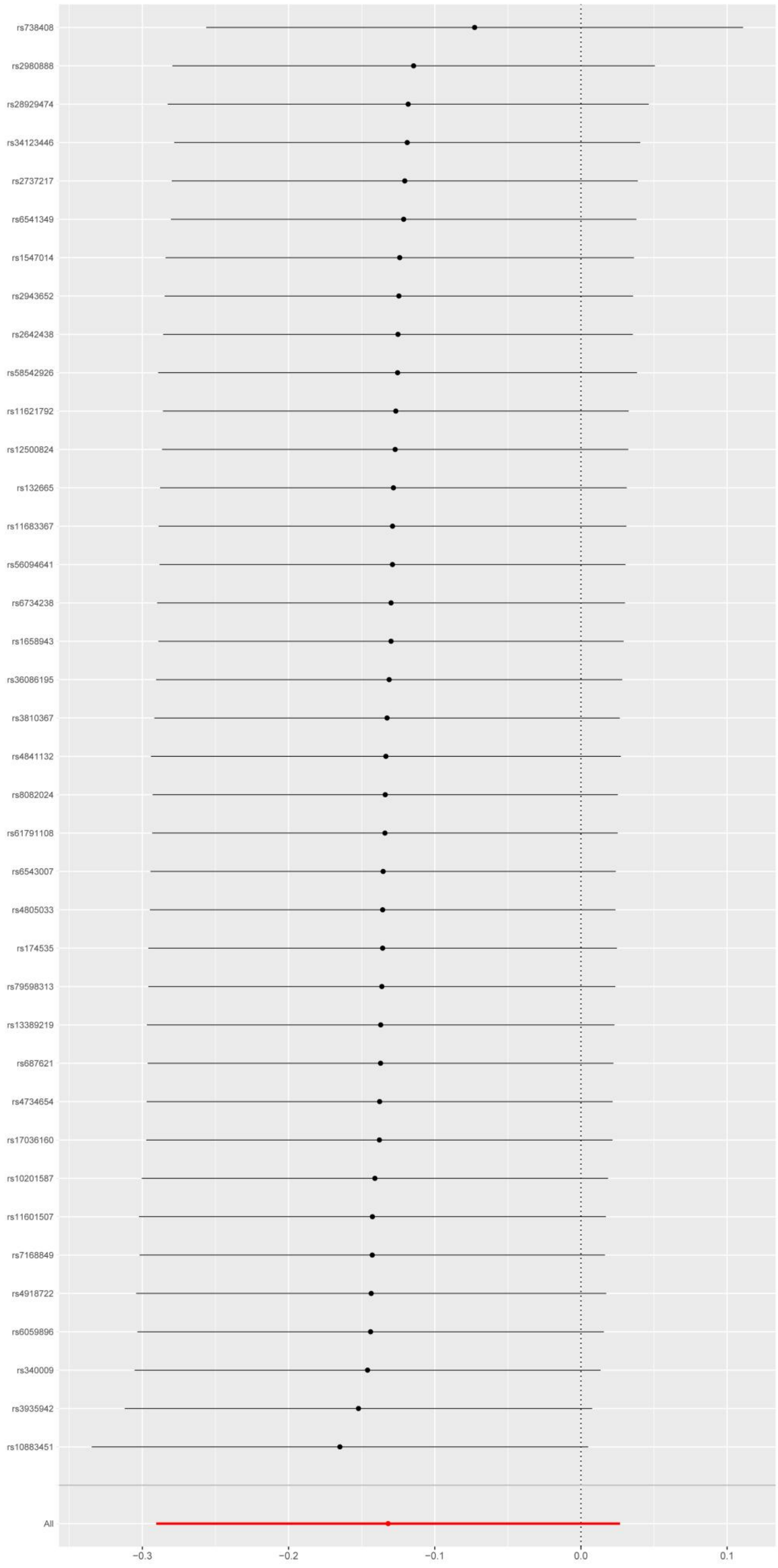

Figure S8. Leave-one-out analysis for liver fibrosis and cirrhosis and AD

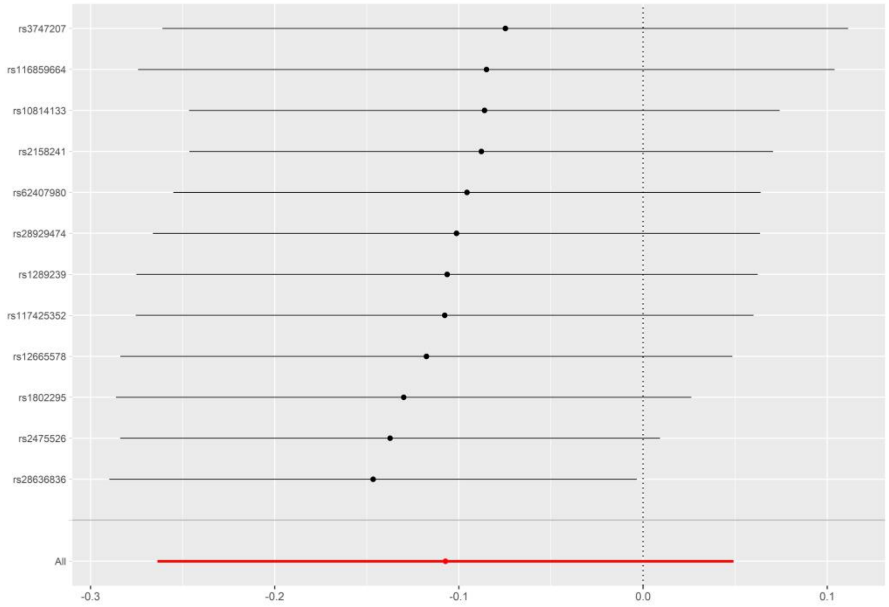

Figure S9. Leave-one-out analysis for MASLD (based on cALT) and VD

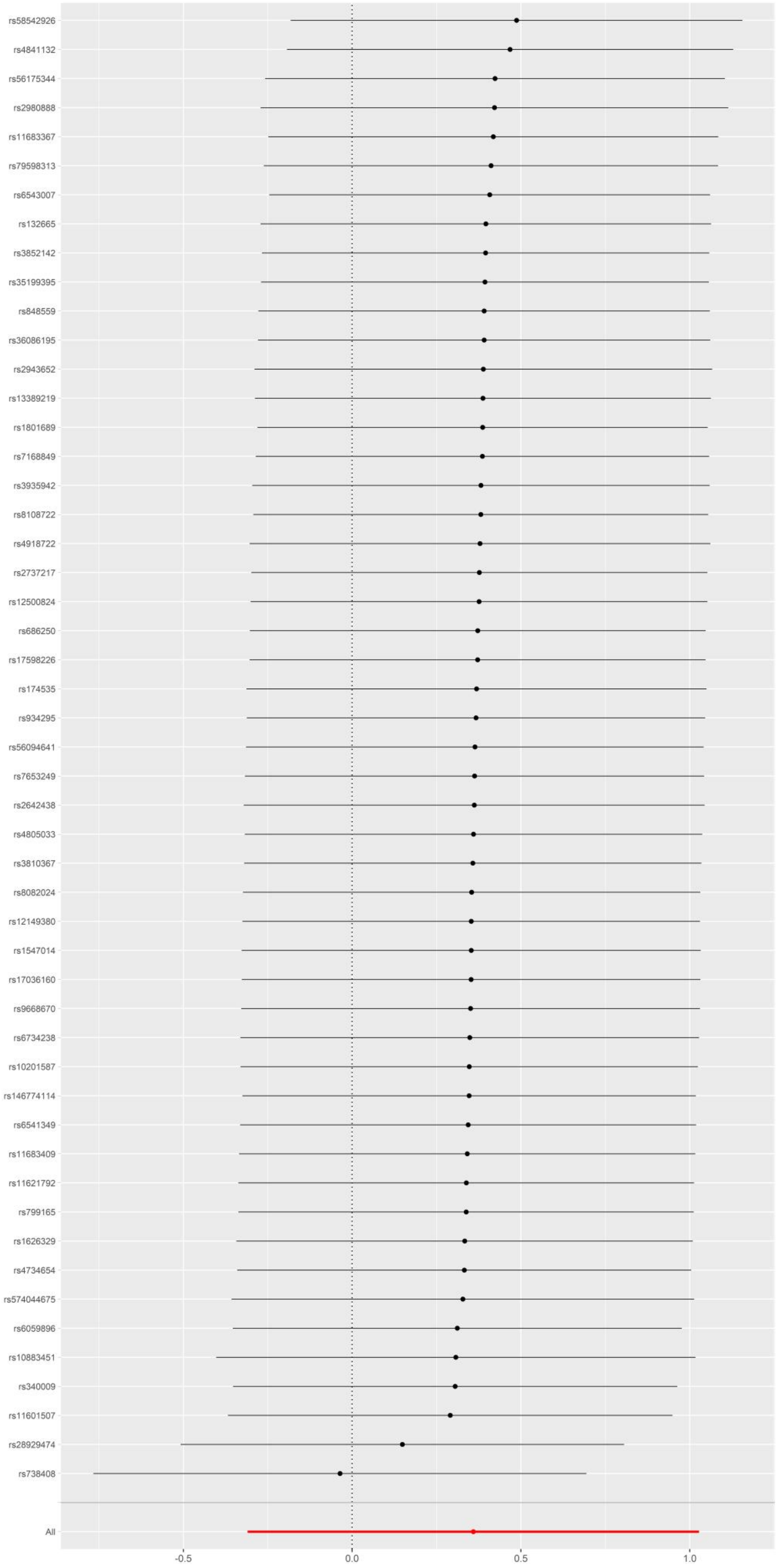

Figure S10. Leave-one-out analysis for MASH and VD

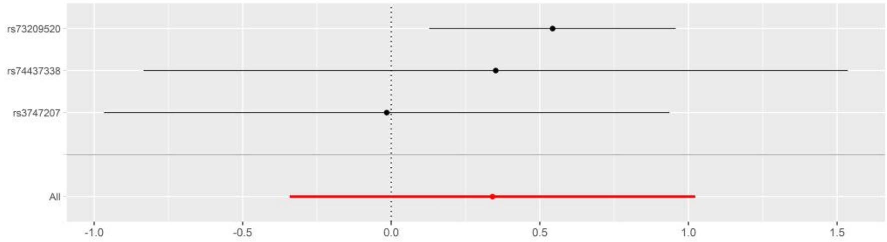

Figure S11. Leave-one-out analysis for liver fibrosis and cirrhosis and VD

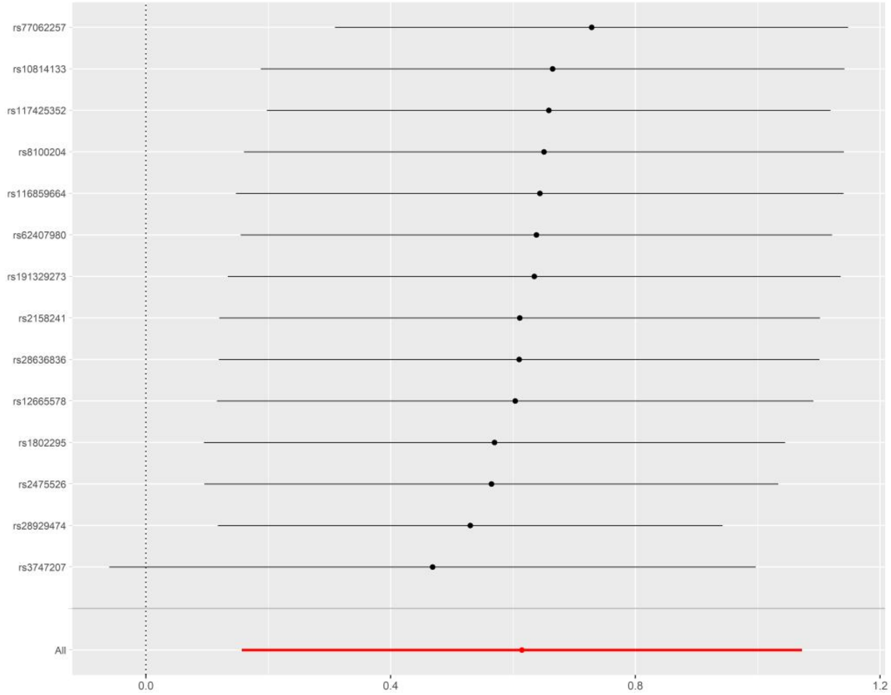

Figure S12. Leave-one-out analysis for MASLD (based on cALT) and DLB

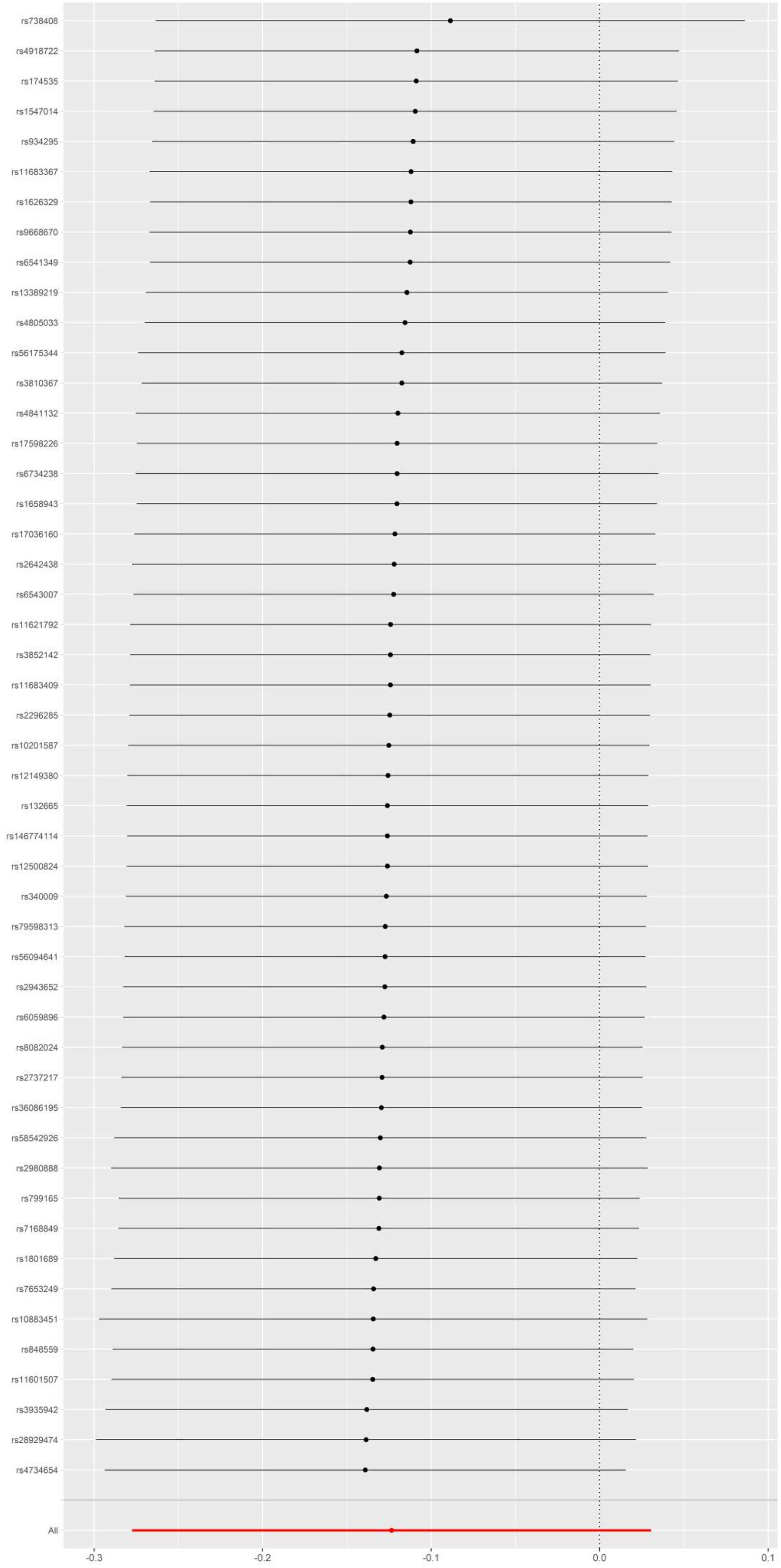

Figure S13. Leave-one-out analysis for MASH and DLB

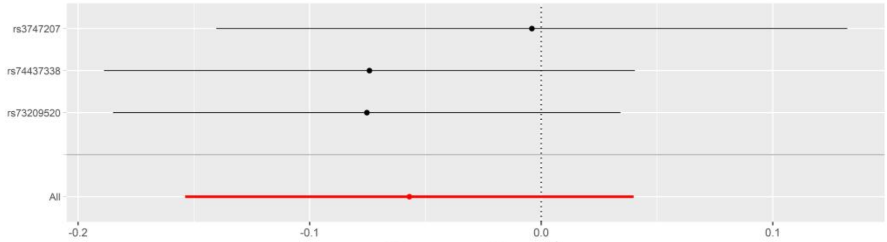

Figure S14. Leave-one-out analysis for liver fibrosis and cirrhosis and DLB

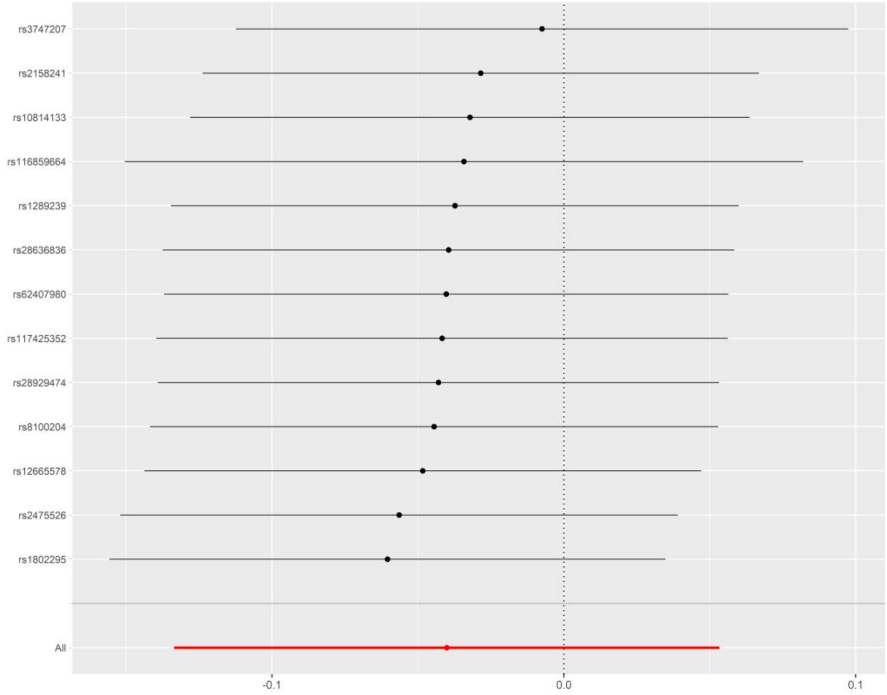

Figure S15. Leave-one-out analysis for MASLD (based on cALT) and FTD

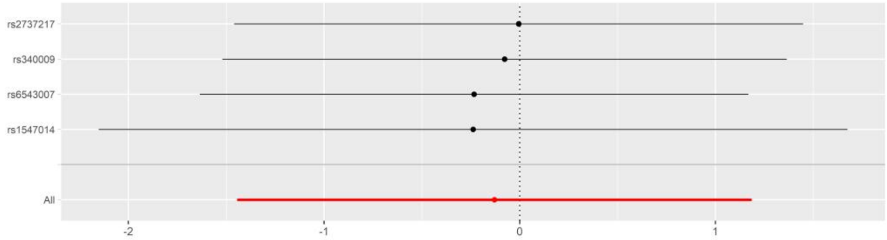

Figure S16. Leave-one-out analysis for MASLD (based on image-supported cALT) and AD

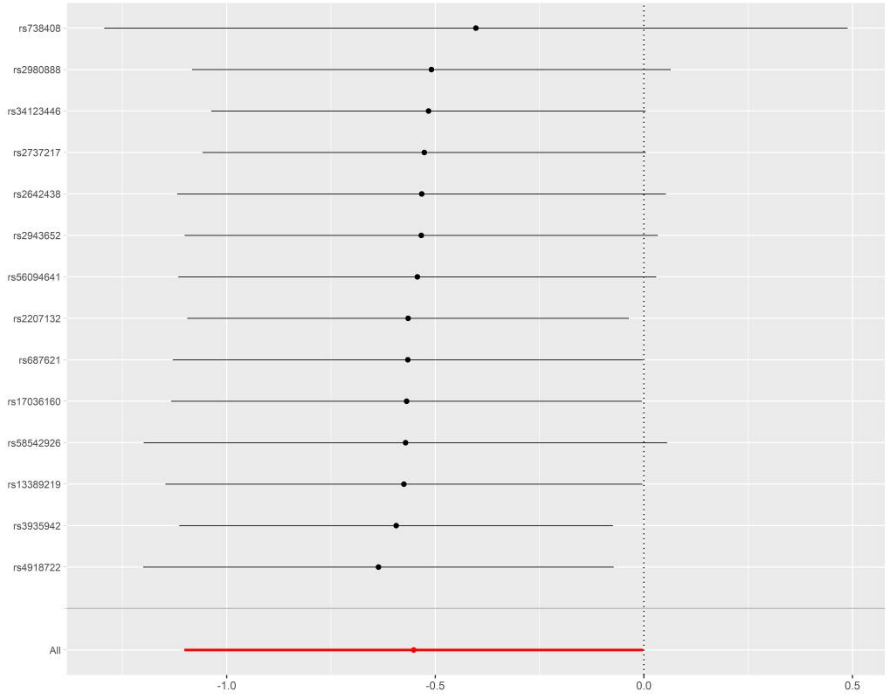

Supplement: S1 File — (PDF) [file pone.0297883.s001.pdf]
